# Film dressing – A versatile no-mess operative field around the head and neck

**DOI:** 10.1016/j.jpra.2019.10.006

**Published:** 2019-11-09

**Authors:** Harshul Dev Measuria, Zita Maria Jessop, Robert Thomas Duncan, Nicholas Wilson-Jones

**Affiliations:** Welsh Centre for Burns & Plastic Surgery, Morriston Hospital, Swansea Bay University Health Board, Heal Maes Eglwys, Morriston, Swansea SA6 6NL, United Kingdom

**Keywords:** Hair, Interference, Ear, Film, Tegaderm

*Dear Sir*,

Hair interference in ear and scalp surgery can be a nuisance. This is particularly true during pinnaplasty, where post-auricular access during placement of Mustarde and Furnas sutures can be made even more difficult by the interference of stray hairs.^1^^,^^2^

Different techniques have been described to help overcome the problem of hair interference such as traditional surgical drapes or stockinet^3^^,^^4^ tubigrip and gauze.^2^ However, waterproofing the hair from blood remains an issue. The use of a latex or silicone swim cap^1^ and haemorrhoid bands^5^ have also been described for ear and scalp surgery respectively, but these may not always be to hand in the operating theatre. Gels and preparatory agents can be messy and contribute to the sub-bandage itch.^1^

The authors would like to present an alternative method of tackling the hair interference/hair staining problem when approaching pinnaplasty with added advantages of keeping the drapes in position.

We use 3M™ Tegaderm™ 10 × 12 cm dressings for this procedure however other film dressings could be used. A single sheet of film dressing should be shaped and applied after prepping the area. The dressing should be folded in half on the short axis and a triangular wedge cut out two thirds along the crease - the depth should correspond to 50% of the height of the patient's ear.

Once cut, the paper window is removed to expose the adherent side before placing the ear through the hole in the dressing - the outer paper frame should be left in situ to allow the dressing to hold it's shape and prevent the edges from curling. Eccentric placement to ensure coverage posteriorly and cranially to encompass a greater proportion of the hairline is preferred; the head may need to be rotated slightly for application.

Figures 1 and 2 illustrate the preparation of a Tegaderm™ and final placement of the dressing in situ in preparation to operate. The Tegaderm™ is easily removed at the end of the procedure with minimal trauma to hair.

**Figure 1:**
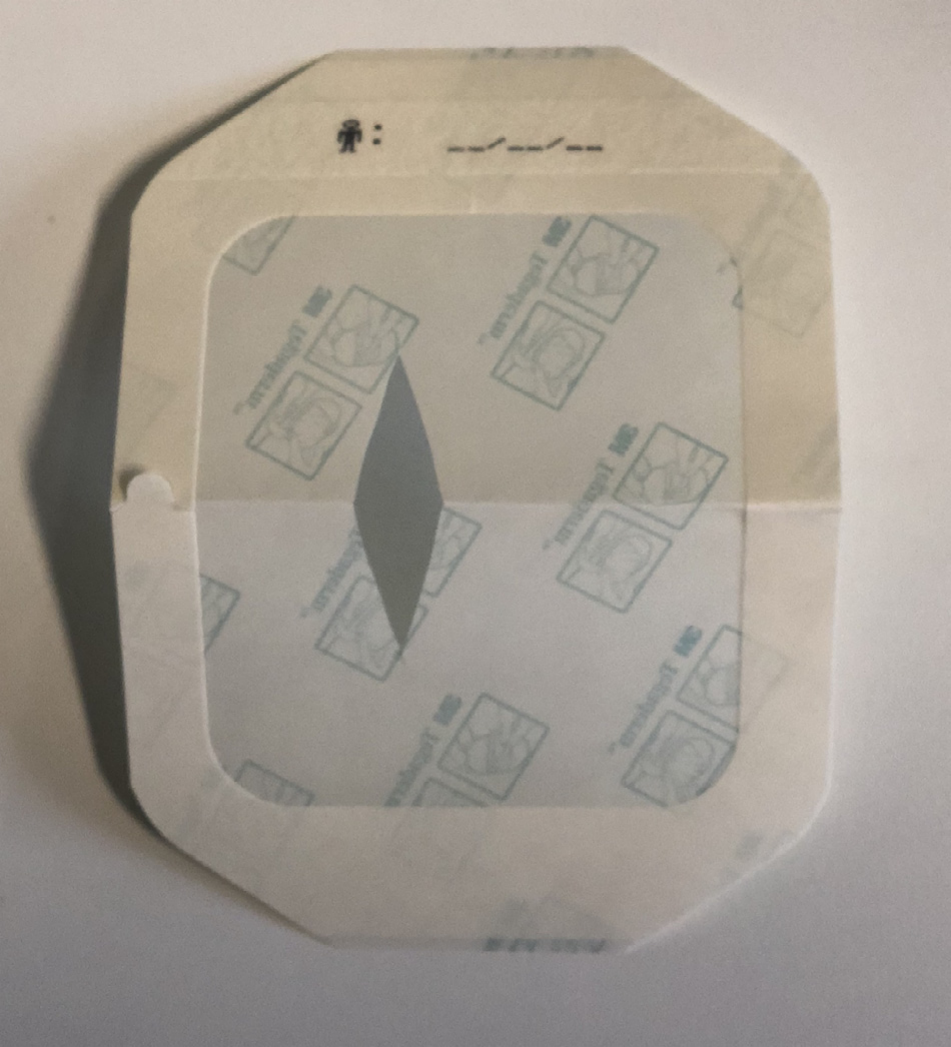
a) Film dressing prepared in half b) Cut a chevron two thirds2/3 along the folded edge c) Dressing with chevron removed d) Final dressing position prior to application.

**Figure 2:**
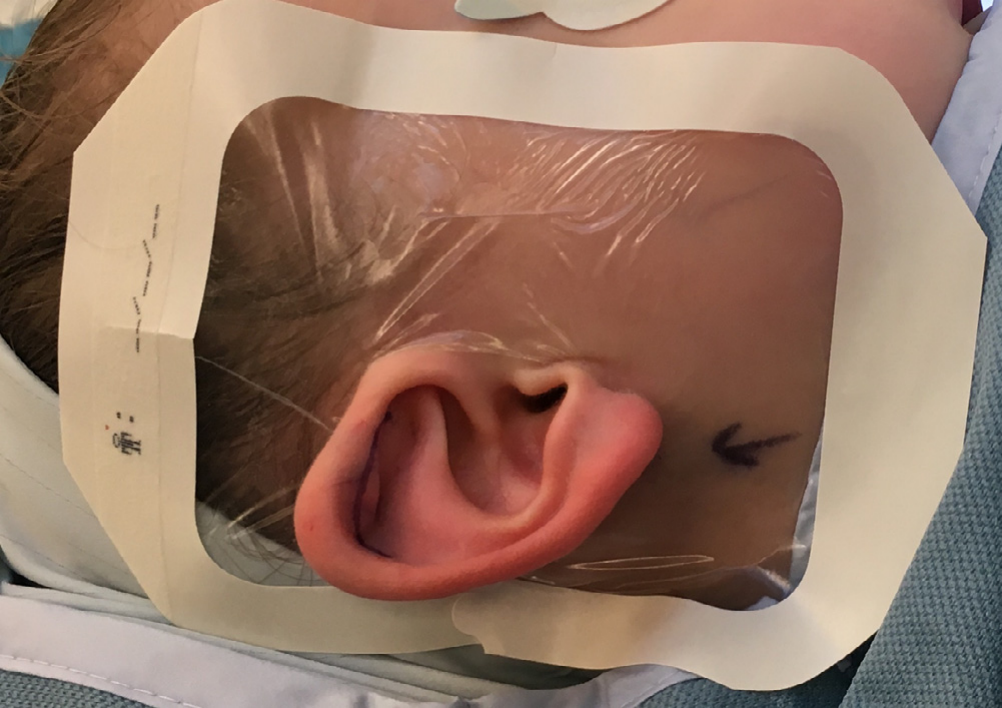
Film dressing applied in preparation to operate.

In our experience, the cost effective (£0.37/dressing) use of a film dressing during pinnaplasty has addressed the issue of hair interference, reducing the need to cut the hair as well as leaving patients without blood staining post-operatively.

## Funding

None

## Conflicts of interest

None declared
